# Fatal *Chlamydia avium* Infection in Captive Picazuro Pigeons, the Netherlands

**DOI:** 10.3201/eid2610.191412

**Published:** 2020-10

**Authors:** Marja Kik, Marloes Heijne, Jooske IJzer, Guy Grinwis, Yvonne Pannekoek, Andrea Gröne

**Affiliations:** Utrecht University, Utrecht, the Netherlands (M. Kik, J. IJzer, G. Grinwis, A. Gröne);; Wageningen Bioveterinary Research, Lelystad, the Netherlands (M. Heijne);; University of Amsterdam, Amsterdam, the Netherlands (Y. Pannekoek)

**Keywords:** chlamydia, Chlamydia avium, pigeons, Picazuro pigeons, real-time PCR, bacteria, Patagioenas picazuro, the Netherlands, aviaries, zoonoses

## Abstract

In 2016, an outbreak of *Chlamydia avium* infection occurred among Picazuro pigeons (*Patagioenas picazuro*) living in an aviary in the Netherlands. Molecular typing revealed a unique strain of *C. avium*. Our findings show that *C. avium* infection, which usually causes subclinical infection, can cause fatal disease in pigeons.

Until approximately 2014, *Chlamydia psittaci* was the only *Chlamydia* species detected in birds. Researchers have catalogued »465 bird species affected by this pathogen, which mainly causes subclinical infections but sometimes results in acute disease and death (*1*). In humans, *C. psittaci* is highly infectious and can cause severe pneumonia. *Chlamydia* bacteria, which are present in (dried) excreta or feather dust, are transmitted through direct contact or inhalation. In 2014, researchers proposed 2 new members of *Chlamydiaceae*: *C. avium* and *C. gallinacea* (*2*). *C. avium* affects pigeons and psittacine birds, whereas *C. gallinacea* affects poultry. Most *C. avium* and *C. gallinacea* infections in birds are subclinical, and the zoonotic potential of these species is unknown (*3*).

In 2016, an outbreak of *C. avium* infection occurred among 11 Picazuro pigeons (*Patagioenas picazuro*) housed in an aviary with other bird species in the Netherlands. The birds lost weight, had ruffled feathers, and were anorexic. Despite treatment with fluids, force-feeding, and in 1 bird, doxycycline treatment (50 mg/kg 1×/d), all 11 animals died or were euthanized. Necropsy revealed that 9 of these birds were in poor physical condition, lacking fat and pectoral muscle mass. The livers and spleens were enlarged; the livers extended an average of 0.5 cm beyond the rear edge of the sternum, whereas the mean diameter of the spleens was 1.0 cm, approximately twice as large as the normal size. We suspected *Chlamydia* infection because of intracellular inclusions in Stamp (modified Ziehl Neelsen)–stained cytology of liver and spleen. We found multifocal heterophilic and lymphoplasmacytic infiltrates with necrosis in the liver and lymphoid depletion with necrosis and heterophilic infiltrates in the spleen. We stained slides with polyclonal antibodies against *Chlamydia* (bioMérieux, https://www.biomerieux.com) after a standard Avidin Biotin Complex protocol (*4*); liver and kidney tissues from 7 birds tested positive for *Chlamydia*. We did not observe any histologic changes consistent with viral inclusions or bacterial infection.

Because psittacosis in birds is a notifiable disease in the Netherlands, we informed public health authorities of our results. We forwarded frozen tissue samples to the Wageningen Bioveterinary Research institute to confirm *C. psittaci* infection. We also collected and forwarded 2 Picazuro pigeon carcasses and 3 pooled fecal samples from contact birds (i.e., Roseate spoonbill [*Platalea ajaja*], Puna ibis [*Plegadis ridgwayi*], and Scarlet ibis [*Eudocimus ruber*]), from the aviary. Two liver samples, 2 conjunctival and cloacal swabs, and 3 pooled fecal samples initially tested negative for *C. psittaci*, *C. abortus*, *C. felis*, and *C. caviae* in a PCR selective for the *omp*A gene. Because the liver and kidney samples of 7 pigeons tested positive for antibodies against *Chlamydia*, we submitted samples from all 11 pigeons and the 3 pooled fecal samples for further testing with real-time PCR selective for the 23S gene of *Chlamydiaceae* (*5*) and a duplex real-time PCR selective for *C. gallinacea* and *C. avium* (*3*,*6*). All 11 pigeons tested positive for *C. avium* in >1 samples of conjunctiva, cloaca, liver or intestines. The pooled fecal samples of contact birds tested negative in a PCR for *Chlamydiaceae* (Appendix).

We used Buffalo green monkey cells to isolate *Chlamydia* from the spleen of 1 of the pigeons that tested positive. Multilocus sequence typing using the concatenated sequences of 7 housekeeping genes revealed that this isolate is a unique sequence type, 254, that is closely related to the other 3 *C. avium* strains previously described (*2*) (Figure).

**Figure:**
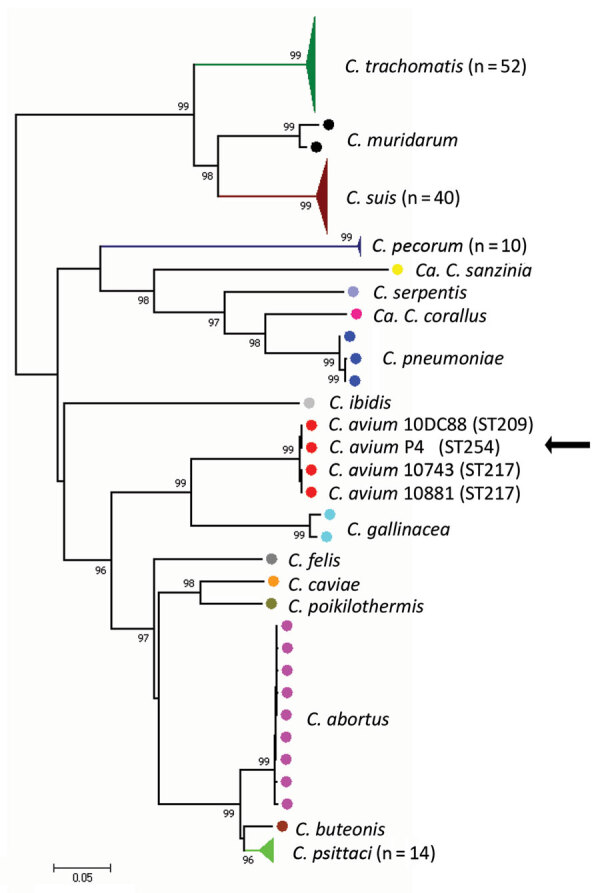
Phylogenetic analyses of concatenated sequences of 7 housekeeping gene fragments of *Chlamydiaceae*, the Netherlands, 2016. Numbers indicate bootstrap values >90%. Filled circles represent isolates, colored by species. Filled colored triangles represent >9 isolates of the same species; total number of isolates used for the analyses is indicated. The sequence types of the *C. avium* isolates are labeled. *C. avium* isolate P4 is indicated by the arrow. Scale bar indicates sequence divergence. ST, sequence type.

The clinical signs, histopathologic results, and positive intralesional immunohistochemistry findings (Appendix) showed that the birds had generalized disease consistent with a *Chlamydia* infection. Real-time PCR revealed an infection with *C. avium*. Further analysis with multilocus sequence typing showed the isolated strain is unique, but most closely related to other reported *C. avium* strains. *C. avium* has been detected mainly in urban or feral pigeons without clinical signs and in co-infections of feral pigeons with *C. psittaci* (*2*). 

Our results show that *C. avium* strains might also cause severe, potentially fatal infections in birds. Data on *C. avium* are limited, but several factors might explain the severity of the clinical signs. Unlike previously reported cases, these pigeons were held in captivity. Furthermore, we cannot exclude possible differences in virulence between sequence types of *C. avium*. No human cases were reported during this outbreak, so the zoonotic potential of *C. avium* remains unknown.

Appendix Additional information about fatal *Chlamydia avium* infection in captive Picazuro pigeons, the Netherlands.
