# Supplementary material for: Fatal Chlamydia avium Infection in Captive Picazuro Pigeons, the Netherlands
Source: Emerg Infect Dis. 2020 Oct;26(10):2520–2. doi: 10.3201/eid2610.191412 (PMC7510720; doi:10.3201/eid2610.191412)
Supplement: Appendix — Additional information about fatal Chlamydia avium infection in captive Picazuro pigeons, the Netherlands. [file 19-1412-Techapp-s1.pdf]

Article DOI: https://doi.org/10.3201/eid2610.191412

Fatal *Chlamydia avium* Infection in Captive Picazuro Pigeons, the Netherlands

Appendix

Strain Isolation and Propagation

Buffalo green monkey (BGM) cells were seeded with Dulbecco's Modified Eagle Medium (DMEM, GIBCO, Life Technologies Limited, UK) and 10% serum in 6-well plates and 24-well plates (Greiner Bio-One GmbH, Germany) with glass coverslips. The plates were incubated at 37°C with 5% CO_2 in a humidified incubator until an 80% confluent monolayer was achieved. For the primary inoculation a 10% suspension of positive spleen material in sucrose phosphate glutamate was used. After inoculation, the plates were centrifuged at 2450 × g and 37°C for 60 min and subsequently incubated for 2 h. The medium was then replaced with UltraMDCK serum-free medium (Lonza, USA) (*1*).

After 4 days of incubation, the plates with glass coverslips were fixed with ice cold methanol and stained with the IMAGEN Chlamydia (Oxoid Ltd, UK) immunofluorescence kit for detection of *Chlamydia* inclusions. The 6-well plate was used for further passaging the strain.

Chlamydia Harvesting and DNA Isolation

Genomic DNA was prepared from 6-well plates of the fourth and fifth passage of our isolated strain. Plates were freeze-thawed twice, and the cells were subsequently harvested. This suspension was centrifuged for 10 minutes at 500 × g to remove cell debris. The supernatant was filtered through a 0.8 µm filter and centrifuged at 30,000 × g. The supernatant was discarded, and the pellet was suspended in DPBS (Gibco, Life Technologies Limited) with 0.05% Tween-20. This centrifuge step was repeated twice. At the last step the pellet was suspended in 500 µL fresh DNase and RNase and incubated for 3 h at 37°C. After incubation, 25 mL of PBS with 0.05% Tween-20 was added and the suspension was again centrifuged 30,000 × g. The pellet was subjected to overnight digestion with proteinase K and tissue lysis buffer ATL (Qiagen

GmbH, Germany) at 56°C and DNA was isolated according to the DNeasy Blood and Tissue kit (Qiagen GmbH, Germany).

Genome Sequencing, Assembly, and Phylogenetic Analyses

The DNA samples were prepared for Illumina sequencing using the SMARTer® ThruPLEX® DNA-Seq kit (Takara Bio, USA) according to the manufacturer's protocol. Quality control of the library preparation was performed on a Tapestation 2200 (Agilent Technologies, Germany) and the DNA concentration was determined on a Clariostar (BMG Labtech, the Netherlands) with use of the Quant-IT PicoGreen® dsDNA kit (Invitrogen Ltd, UK). Sequencing was performed on an Illumina MiSeq platform. The complete genome and plasmid sequences were assembled using SPAdes 3.9 (*2*). The genome size (coverage 73-fold) was determined to be 1,041,128 bp, the plasmid size (coverage 79-fold) 7,640 bp. The sequences are deposited in the publicly available Bacterial Isolate Genome Sequence Database (BIGSdb) ((http://pubmlst.org/chlamydiales) (id: 4411). Phylogenetic analyses of concatenated sequences were carried out in MEGA6 (*3*). A phylogenetic tree was constructed using the neighbor-joining algorithm using maximum composite likelihood model. Bootstrap test was for 500 repetitions. Strains and sequence types are displayed in the Appendix Table.

References

1. Sachse K, Laroucau K, Riege K, Wehner S, Dilcher M, Creasy HH, et al. Evidence for the existence of two new members of the family *Chlamydiaceae* and proposal of *Chlamydia avium* sp. nov. and *Chlamydia gallinacea* sp. nov. Syst Appl Microbiol. 2014;37:79–88. PubMed https://doi.org/10.1016/j.syapm.2013.12.004

2. Bankevich A, Nurk S, Antipov D, Gurevich AA, Dvorkin M, Kulikov AS, et al. SPAdes: a new genome assembly algorithm and its applications to single-cell sequencing. J Comput Biol. 2012;19:455–77. PubMed https://doi.org/10.1089/cmb.2012.0021

3. Tamura K, Stecher G, Peterson D, Filipski A, Kumar S. MEGA6: Molecular Evolutionary Genetics Analysis version 6.0. Mol Biol Evol. 2013;30:2725–9. PubMed https://doi.org/10.1093/molbev/mst197

Appendix Table. Characteristics of *Chlamydia avium* isolates*

ID	Isolate	Species	Host	Country	*gat*A	*opp*A	*hfl*X	*gid*A	*eno*A	*hem*N	*fum*C	Sequence type	No.
4429	G3/2742-324	*Ca. Chlamydia corallus*		Switzerland	79	73	84	86	90	70	72	265	1
4428	2742-308	*Ca. Chlamydia sanzinia*		Switzerland	84	77	89	91	94	73	77	268	1
2	T20	*Chlamydia abortus*	Sheep	UK	5	8	6	8	8	4	5	19	
890	AB7	*Chlamydia abortus*	Sheep	France	18	8	6	8	8	14	5	25	
4281	C21_98	*Chlamydia abortus*	Goat	Namibia	5	8	6	8	8	4	18	29	
62	POS	*Chlamydia abortus*	Sheep		13	18	6	19	16	4	17	30	
4346	162STDY5437296	*Chlamydia abortus*	Sheep		5	8	6	8	8	14	5	86	
4276	C1_98	*Chlamydia abortus*	Sheep	Germany	5	30	6	8	8	4	5	148	
4284	99DC1	*Chlamydia abortus*	Sheep	Germany	5	31	6	41	8	4	5	149	
4293	10DC83	*Chlamydia abortus*	sheep	Germany	5	8	6	41	8	4	5	150	8
300	10DC88	*Chlamydia avium*	Psittacine bird	Germany	57	54	63	66	69	47	49	209	
879	10_743_SC13	*Chlamydia avium*	Pigeon		58	55	67	68	69	51	49	217	
880	10_881_SC42	*Chlamydia avium*	Pigeon		58	55	67	68	69	51	49	217	
4411	C. avium P4	*Chlamydia avium*	Pigeon	The Netherlands	57	54	63	68	69	47	49	254	4
211	GPIC	*Chlamydia caviae*	Guinea pig		6	9	7	9	9	5	6	20	1
891	Fe/C-56	*Chlamydia felis*	Cat		7	10	8	10	10	50	7	214	1
3984	JX-1	*Chlamydia gallinacea*	Chicken	China	44	35	38	45	36	29	28	155	
888	08-1274/3	*Chlamydia gallinacea*	Chicken	France	44	36	39	45	36	30	28	156	2
4409	10_1398_11	*Chlamydia ibidis*	Ibis	France	40	74	64	67	70	48	50	264	1
45	Nigg	*Chlamydia muridarum*	Mouse		8	11	9	11	11	7	8	22	
872	MopnTet14	*Chlamydia muridarum*	Mouse		8	11	9	11	11	49	8	210	2
209	E58	*Chlamydia pecorum*	Cattle		9	12	10	12	12	8	9	23	
876	VR629	*Chlamydia pecorum*	Sheep	USA	20	12	20	22	18	17	9	48	
181	L71	*Chlamydia pecorum*	Pig		22	21	10	23	20	8	20	50	
175	L1	*Chlamydia pecorum*	Pig	Australia	21	22	22	24	20	8	9	52	
185	L17	*Chlamydia pecorum*	Pig		23	22	23	24	21	8	9	53	
288	W73	*Chlamydia pecorum*	Sheep		31	12	10	29	18	8	9	68	
875	MC/MarsBar	*Chlamydia pecorum*	Koala	Australia	27	12	10	23	23	8	9	69	
810	PV3056/3	*Chlamydia pecorum*	Cow		31	12	35	24	30	8	20	205	
809	P787	*Chlamydia pecorum*	Sheep		37	12	10	36	18	8	9	206	
874	IPTaLE	*Chlamydia pecorum*	Koala	Australia	21	12	10	23	23	8	9	211	10
29	CM-1	*Chlamydia pneumoniae*	Human		4	7	5	6	5	3	4	16	
32	CWL-011	*Chlamydia pneumoniae*	Human		4	7	5	7	6	3	4	17	
206	LPCoLN	*Chlamydia pneumoniae*	Koala		19	19	66	21	17	16	4	215	
4427	S15-834K	*Chlamydia poikilothermis*	Snake	Switzerland	83	76	88	90	93	72	76	267	n =3
192	6BC	*Chlamydia psittaci*	Parakeet		10	13	11	13	13	9	10	24	
197	CP3	*Chlamydia psittaci*	Pigeon		11	14	15	14	13	9	11	27	
198	GR9	*Chlamydia psittaci*	Mallard		12	13	11	13	13	9	12	28	
199	M56	*Chlamydia psittaci*	Muskrat		17	17	17	18	13	12	16	31	
194	WC	*Chlamydia psittaci*	Cow		12	13	13	13	13	13	10	32	
200	MN	*Chlamydia psittaci*	Human		11	14	15	14	13	11	13	35	

ID	Isolate	Species	Host	Country	*gat*A	*opp*A	*hfl*X	*gid*A	*eno*A	*hem*N	*fum*C	Sequence type	No.
95	84_2334	*Chlamydia psittaci*	Yellow-crowned amazon parrot		13	15	12	15	14	4	14	36	
201	NJ1	*Chlamydia psittaci*	Turkey		15	16	13	16	15	10	15	43	
168	09DC77	*Chlamydia psittaci*	Pigeon		11	14	15	13	13	9	13	47	
195	01DC12	*Chlamydia psittaci*	Pig		11	14	11	14	13	11	13	56	
202	VS225	*Chlamydia psittaci*	Parakeet		12	13	18	13	68	9	10	204	
811	Mat116	*Chlamydia psittaci*	Human	Japan	38	13	11	13	13	9	10	208	
881	99DC5	*Chlamydia psittaci*			10	13	11	13	71	9	10	218	
4359	Zo Pa	*Chlamydia psittaci*	Human	Australia	74	13	11	13	13	9	10	255	14
4426	H15-1957-10C	*Chlamydia serpentis*	Snake	Switzerland	82	75	87	89	92	71	75	266	1
889	MD56	*Chlamydia suis*	Pig	Italy	51	49	52	56	53	40	41	183	
4370	SWA-2	*Chlamydia suis*	Pig	Switzerland	60	50	54	69	72	52	51	219	
4371	4-29b	*Chlamydia suis*	Pig	Switzerland	61	51	55	70	73	53	52	221	
4403	9-1b	*Chlamydia suis*	Pig	Switzerland	63	59	70	71	75	55	54	223	
4372	3-25b	*Chlamydia suis*	Pig	Switzerland	62	58	69	70	74	54	53	224	
4374	5-27b	*Chlamydia suis*	Pig	Switzerland	46	60	69	72	76	37	55	225	
4375	2-26b	*Chlamydia suis*	Pig	Switzerland	64	59	71	71	75	55	56	226	
4376	5-22b	*Chlamydia suis*	Pig	Switzerland	63	59	72	71	77	56	57	227	
4377	SWA-14	*Chlamydia suis*	Pig	Switzerland	65	61	73	70	74	57	58	228	
4378	SWA-86	*Chlamydia suis*	Pig	Switzerland	66	58	74	70	74	54	59	229	
4379	3-29b	*Chlamydia suis*	Pig	Switzerland	63	59	75	71	75	55	57	230	
4380	19-23a	*Chlamydia suis*	Pig	Switzerland	67	62	76	73	78	58	60	231	
4381	30-22b	*Chlamydia suis*	Pig	Switzerland	68	63	73	74	79	53	52	232	
4382	17-23b	*Chlamydia suis*	Pig	Switzerland	69	61	73	70	80	57	61	233	
4383	9-1a	*Chlamydia suis*	Pig	Switzerland	64	59	70	75	77	55	54	234	
4385	9-29b	*Chlamydia suis*	Pig	Switzerland	63	59	78	71	77	55	57	236	
4386	10-26b	*Chlamydia suis*	Pig	Switzerland	63	59	80	71	75	55	54	237	
4388	1-25b	*Chlamydia suis*	Pig	Switzerland	66	64	73	77	74	60	63	238	
4389	1-28a	*Chlamydia suis*	Pig	Switzerland	64	59	70	71	75	55	57	239	
4390	11-17b	*Chlamydia suis*	Pig	Switzerland	69	65	69	78	81	58	55	240	
4391	1-28b	*Chlamydia suis*	Pig	Switzerland	70	66	73	70	73	61	64	241	
4392	22-22b	*Chlamydia suis*	Pig	Switzerland	68	58	69	79	82	62	55	242	
4393	8-29b	*Chlamydia suis*	Pig	Switzerland	66	57	68	70	83	53	65	243	
4394	8-17a	*Chlamydia suis*	Pig	Switzerland	71	67	77	80	84	63	43	244	
4396	15-27b	*Chlamydia suis*	Pig	Switzerland	72	68	43	80	74	64	59	245	
4397	17-23a	*Chlamydia suis*	Pig	Switzerland	67	64	73	70	74	37	66	246	
4398	1-25a	*Chlamydia suis*	Pig	Switzerland	73	64	74	76	74	65	37	247	
4399	14-23b	*Chlamydia suis*	Pig	Switzerland	63	69	70	71	75	37	55	248	
4400	6-17a	*Chlamydia suis*	Pig	Switzerland	64	59	78	81	75	66	57	249	
4401	19-23b	*Chlamydia suis*	Pig	Switzerland	66	70	79	82	85	63	66	250	
4384	9-25a	*Chlamydia suis*	Pig	Switzerland	53	64	49	76	74	59	62	251	
4387	3-25a	*Chlamydia suis*	Pig	Switzerland	66	64	45	76	74	65	63	252	
4413	R22	*Chlamydia suis*	Pig	USA	75	71	81	83	87	67	67	256	
4412	R19	*Chlamydia suis*	Pig	USA	63	59	70	71	86	49	57	257	
4415	R27	*Chlamydia suis*	Pig	USA	76	71	81	83	87	67	67	258	
4416	H5	*Chlamydia suis*	Pig	USA	77	59	70	71	75	68	54	259	

ID	Isolate	Species	Host	Country	*gat*A	*opp*A	*hfl*X	*gid*A	*eno*A	*hem*N	*fum*C	Sequence type	No.
4417	H7	*Chlamydia suis*	Pig	USA	78	72	82	84	54	69	68	260	
4418	S45	*Chlamydia suis*	Pig	Austria	63	59	70	71	88	66	69	261	
4419	Rogers132	*Chlamydia suis*	Pig	USA	63	69	78	85	75	49	70	262	
4420	R1	*Chlamydia suis*	Pig	USA	75	71	83	83	89	67	71	263	40
66	A/HAR-13	*Chlamydia trachomatis*	Human	Saudi Arabia	3	3	4	5	3	2	3	3	
234	E/150	*Chlamydia trachomatis*	Human		3	1	1	2	4	2	3	4	
246	G/SotonG1	*Chlamydia trachomatis*	Human	UK	3	3	2	5	3	1	3	6	
727	ERR111579	*Chlamydia trachomatis*	Human		2	1	1	2	4	2	3	8	
858	J/6276tet1	*Chlamydia trachomatis*	Human		3	3	2	4	3	2	3	9	
252	L1/440/LN	*Chlamydia trachomatis*	Human	USA	1	3	3	3	2	2	2	11	
101	ERR175646	*Chlamydia trachomatis*	Human		3	4	1	2	4	2	3	12	
215	D/UW-3/CX	*Chlamydia trachomatis*	Human	USA	3	3	2	5	3	2	3	13	
99	D(s)2923	*Chlamydia trachomatis*	Human		3	1	2	2	4	2	3	38	
480	ERR111627	*Chlamydia trachomatis*	Human		3	3	19	4	3	2	3	42	
79	L2/434/Bu	*Chlamydia trachomatis*	Human	USA	1	3	3	3	2	2	19	44	
227	B/Jali20/OT	*Chlamydia trachomatis*	Human	The Gambia	3	3	4	20	3	2	3	45	
96	F(s)/70	*Chlamydia trachomatis*	Human		2	1	1	2	3	2	3	91	
190	ERR189729	*Chlamydia trachomatis*	Human		3	3	28	5	3	18	3	92	
226	ERR111630	*Chlamydia trachomatis*	Human		3	1	29	2	4	2	3	93	
235	E/Bour	*Chlamydia trachomatis*	Human	USA	3	4	1	32	4	2	3	94	
243	G/11222	*Chlamydia trachomatis*	Human		3	3	28	5	3	2	3	95	
487	ERR164678	*Chlamydia trachomatis*	Human		3	3	2	1	3	1	3	96	
491	ERR164676	*Chlamydia trachomatis*	Human		33	1	2	2	4	2	3	97	
499	ERR164657	*Chlamydia trachomatis*	Human		1	3	30	3	4	2	19	98	
509	ERR189730	*Chlamydia trachomatis*	Human		3	3	31	5	3	2	3	99	
516	ERR026580	*Chlamydia trachomatis*	Human		3	3	2	5	3	1	1	100	
532	ERR026583	*Chlamydia trachomatis*	Human		3	3	28	5	3	1	3	101	
565	ERR164646	*Chlamydia trachomatis*	Human		3	3	2	4	24	2	3	102	
592	ERR026548	*Chlamydia trachomatis*	Human		3	1	2	2	4	2	21	103	
615	ERR108273	*Chlamydia trachomatis*	Human		3	2	1	5	4	2	3	104	
701	ERR108274	*Chlamydia trachomatis*	Human		3	2	1	5	4	2	3	104	
616	ERR111622	*Chlamydia trachomatis*	Human		3	3	32	4	3	2	3	105	
620	ERR108299	*Chlamydia trachomatis*	Human		2	3	2	5	3	2	3	106	
624	ERR140806	*Chlamydia trachomatis*	Human		3	4	2	5	4	2	3	107	
632	ERR189744	*Chlamydia trachomatis*	Human		3	3	2	2	3	2	22	108	
636	ERR189754	*Chlamydia trachomatis*	Human		3	25	1	2	4	2	3	109	
646	ERR111593	*Chlamydia trachomatis*	Human		3	1	1	2	3	2	3	110	
684	ERR140824	*Chlamydia trachomatis*	Human		3	1	1	33	4	2	3	111	
687	ERR189742	*Chlamydia trachomatis*	Human		3	3	2	5	3	2	22	112	
695	ERR140813	*Chlamydia trachomatis*	Human		2	3	2	4	3	2	3	113	
699	ERR111555	*Chlamydia trachomatis*	Human		3	1	1	2	4	19	3	114	
738	ERR278143	*Chlamydia trachomatis*	Human		2	1	1	2	4	2	23	115	
749	ERR210969	*Chlamydia trachomatis*	Human		1	3	2	3	2	2	19	116	
759	ERR278220	*Chlamydia trachomatis*	Human		3	3	4	34	3	2	3	117	
771	ERR278181	*Chlamydia trachomatis*	Human		3	2	2	35	4	2	3	118	
787	ERR210990	*Chlamydia trachomatis*	Human		3	3	2	5	25	1	3	119	
806	ERR278135	*Chlamydia trachomatis*	Human		3	1	1	2	26	2	3	120	
812	ERR386222	*Chlamydia trachomatis*	Human		39	1	1	2	4	2	3	121	

ID	Isolate	Species	Host	Country	*gat*A	*opp*A	*hfl*X	*gid*A	*eno*A	*hem*N	*fum*C	Sequence type	No.
842	ERR211021	*Chlamydia trachomatis*	Human		1	3	3	5	2	2	19	122	
850	ERR278184	*Chlamydia trachomatis*	Human		2	2	1	5	4	2	3	123	
862	RC-F(s)/852	*Chlamydia trachomatis*	Human		2	3	3	2	3	2	3	124	
863	RC-F/69	*Chlamydia trachomatis*	Human		1	3	3	2	3	2	19	125	
866	RC-J/953	*Chlamydia trachomatis*	Human		1	3	2	4	3	2	19	126	
868	RC-J/971	*Chlamydia trachomatis*	Human		1	3	3	4	2	2	19	127	
871	RC-L2/55	*Chlamydia trachomatis*	Human		1	3	1	3	2	2	19	128	
626	ERR189735	*Chlamydia trachomatis*	Human		1	3	3	3	4	2	19	207	52
4025	RSHA	*Chlamydia buteonis*	Red-shouldered hawk	USA	56	53	60	64	65	46	47	196	

*Blank spaces indicate not available.

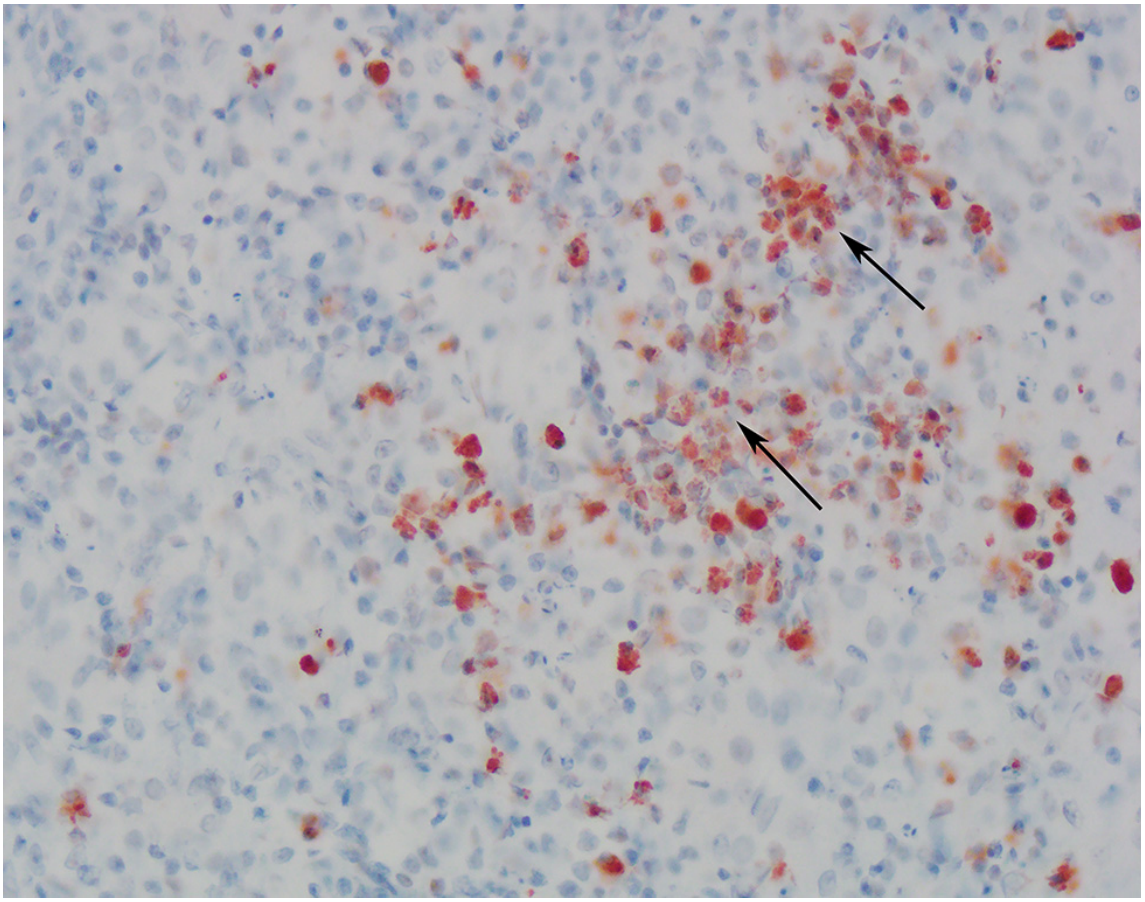

Appendix Figure. Immunohistochemical staining of liver of a pigeon infected with *Chlaymdia avium*, the Netherlands, 2016. Arrows indicate positive immunolabeling for *Chlamydia* antigen. Original magnification ×80.
